# Implications of *Plasmodium vivax* Biology for Control, Elimination, and Research

**DOI:** 10.4269/ajtmh.16-0160

**Published:** 2016-12-28

**Authors:** Piero L. Olliaro, John W. Barnwell, Alyssa Barry, Kamini Mendis, Ivo Mueller, John C. Reeder, G. Dennis Shanks, Georges Snounou, Chansuda Wongsrichanalai

**Affiliations:** 1 UNICEF/UNDP/World Bank/WHO Special Programme on Research and Training in Tropical Diseases (TDR), World Health Organization, Geneva, Switzerland.; 2 Centre for Tropical Medicine and Global Health, Nuffield Department of Medicine, University of Oxford, Oxford, United Kingdom.; 3 Malaria Branch, Division of Parasitic Diseases and Malaria, Center for Global Health, Centers for Disease Control and Prevention, Atlanta, Georgia.; 4 Division of Population Health and Immunity, Walter and Eliza Hall Institute of Medical Research, Melbourne, Australia.; 5 Department of Medical Biology, University of Melbourne, Melbourne, Australia.; 6 Independent Consultant, Colombo 5. Sri Lanka.; 7 Institute of Global Health (ISGLOBAL), Barcelona, Spain.; 8 School of Population Health, University of Queensland, Brisbane, Australia.; 9 Sorbonne Universités, UPMC Univ Paris 06, UPMC UMRS CR7, Paris, France.; 10 Centre d'Immunologie et de Maladies Infectieuses (CIMI)–Paris, Institut National de la Santé et de la Recherche Médicale (INSERM) U1135–Centre National de la Recherche Scientifique (CNRS) ERL 8255, Paris, France.; 11 Independent Scholar, Bangkok, Thailand.

## Abstract

This paper summarizes our current understanding of the biology of *Plasmodium vivax*, how it differs from *Plasmodium falciparum*, and how these differences explain the need for *P. vivax*-tailored interventions. The article further pinpoints knowledge gaps where investments in research are needed to help identify and develop such specific interventions. The principal obstacles to reduce and eventually eliminate *P. vivax* reside in 1) its higher vectorial capacity compared with *P. falciparum* due to its ability to develop at lower temperature and over a shorter sporogonic cycle in the vector, allowing transmission in temperate zones and making it less sensitive to vector control measures that are otherwise effective on *P. falciparum*; 2) the presence of dormant liver forms (hypnozoites), sustaining multiple relapsing episodes from a single infectious bite that cannot be diagnosed and are not susceptible to any available antimalarial except primaquine, with routine deployment restricted by toxicity; 3) low parasite densities, which are difficult to detect with current diagnostics leading to missed diagnoses and delayed treatments (and protracted transmission), coupled with 4) transmission stages (gametocytes) occurring early in acute infections, before infection is diagnosed.

## Introduction

**Figure 1.**
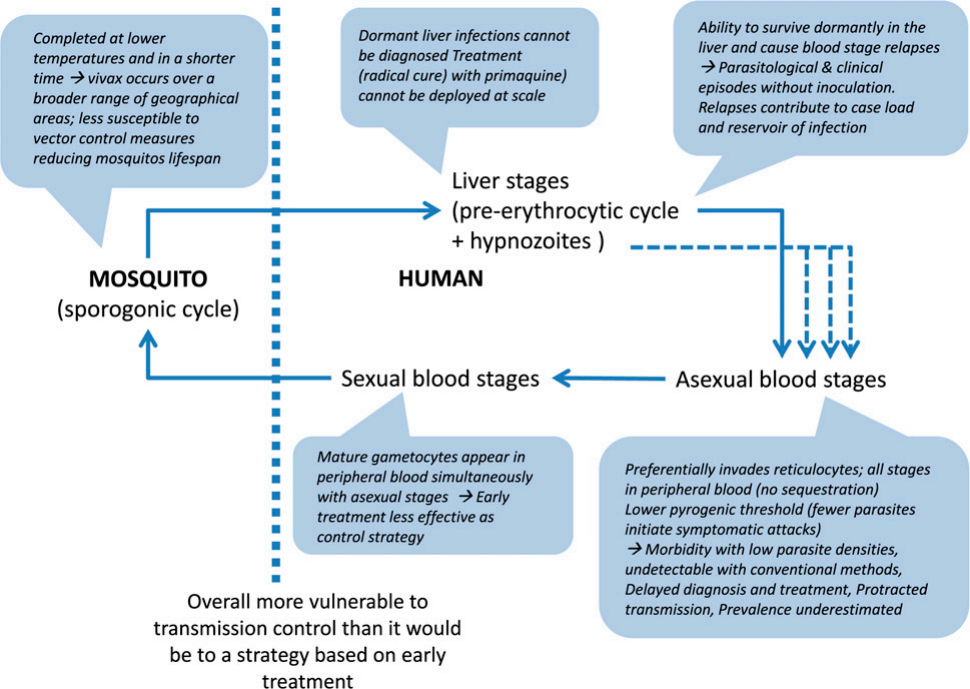
*Plasmodium vivax* cycle and main biological characteristics.

A better understanding of *Plasmodium vivax* biology and how it differs from *Plasmodium falciparum* could help to better target research and interventions that would allow us to intensify control and possibly eliminate vivax malaria. The knowledge and knowledge gaps in *P. vivax* biology are grouped into several intertwined topics: 1) liver stages: hypnozoites and relapses, 2) asexual blood stages and merozoite invasion, 3) asexual blood stages and diagnosis, 4) sexual blood stages and transmission, 5) mosquito stages and vectorial capacity, 6) immunity, 7) pathogenesis, and 8) genetic diversity. Herein, we discuss in turn each of these topics and their implications for the control of *P. vivax* malaria (summarized in Table 1 and Figure 1

).

## Liver Stages: Hypnozoites and Relapses

Persistent quiescent liver forms (hypnozoites) cause relapse infections, generating cases without further sporozoite inoculations by mosquitoes. The latency periods between relapse episodes are strain specific and influenced by the inoculum size, though relapses could also be triggered by external mechanisms, inflammation possibly being one. Radical cure requires the elimination of the hypnozoites. Relapses help maintain the parasite's genetic diversity and reduce its reliance on mosquito transmission for sustenance, hence diminishing the effectiveness of vector control measures.

There are no current diagnostics to detect hypnozoites. Understanding the mechanisms of hypnozoite quiescence and reactivation and developing a safe radical cure are the two most urgent research priorities, but in vitro and in vivo models suitable for biological investigations and drug screening are required to achieve these objectives.^1^

Hypnozoites are evolutionary adaptations present only in the human malaria parasites *P. vivax* and *Plasmodium ovale*, and a few nonhuman primate species, notably *Plasmodium cynomolgi* of macaques, which has long been used as an in vivo model. Histologically, hypnozoites are small, uninucleated parasites that can be visualized by staining with specific antibodies but remain difficult to detect because they are present in low numbers even in hosts given massive inocula.^2^ The biology of these nongrowing, nondividing parasites is largely unknown. Hypnozoites can persist for more than a year in their host cell, the hepatocyte, in a state of reduced metabolic activity, which protects them from all antimalarials except for some 8-aminoquinolines, which presumably act through oxidative stress.

**Figure 2.**
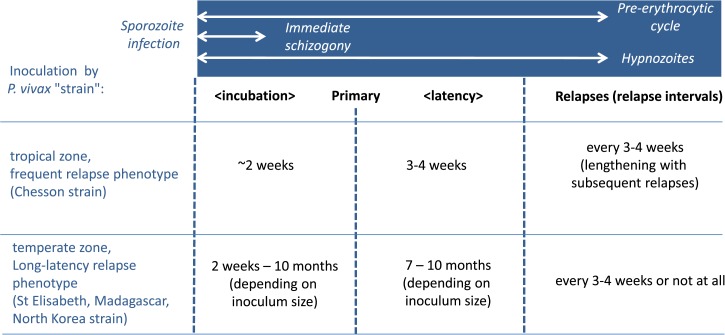
Patterns of primary attack and relapse of the principal *Plasmodium vivax* strains.

The mechanisms regulating hypnozoite dormancy and reactivation remain obscure. Different parasite strains display distinct relapse patterns, ranging from one in which the primary infection occurs several (9–12) months following the infective bite, which is typical of strains from temperate regions including Korean, some Chinese, and the old north European/Russian or “hibernans” strains, to the other extreme where the primary episode occurs 14−21 days after infection followed by a succession of relapses at roughly monthly intervals typified by the tropical Chesson strain from Papua New Guinea (PNG)^3^ (see Figure 2

). Strains midway between these examples with mixed relapse patterns are known and probably frequent, but it is problematic to determine this pattern in endemic regions because distinguishing new infections from relapses is difficult.^4^

It is not ordinarily possible to distinguish primary infections from subsequent relapses. Two subsequent, genetically identical infections could either be from a failure to clear the bloodstream completely of parasites (recrudescence) or activation of a hypnozoite in the liver (relapse). When studying infants with no previous infections, relapses are usually homologous to the initial strain. In most studies, including studies in travelers with time-limited exposures in endemic countries, relapses were found to be genetically distinct^5^,^6^ but related^7^ to either the initial infections or a previous relapse. In drug efficacy studies, it is therefore often possible to distinguish relapses from recrudescence (i.e., if the initial and subsequent parasites are genetically distinct), but not between a relapse and a new infection.

Factors in addition and external to genetic and epigenetic control, such as inflammation, are also likely to trigger hypnozoite activation. Historical and more recent observations suggest that febrile responses from some infections (such as a *P. falciparum*, typhoid fever or relapsing fever) but not others (influenza) could precipitate *P. vivax* relapse.^8^ Toll-like receptors, which activate innate immune responses, could also play a role in hypnozoite activation.^9^ Much more research is needed to decipher the underlying genetic mechanisms and environmental factors that may influence relapse patterns and hypnozoite activation.

*Plasmodium vivax* relapse poses a major challenge to the control and elimination of this species because the multiple episodes can exacerbate the total morbidity resulting from each infection and substantially prolong the duration of the infection. Thus, the potential for transmission onto new hosts, whether infections are symptomatic or asymptomatic, is greatly increased. Understanding the mechanisms of hypnozoite quiescence and reactivation, as well as developing a safe radical cure are the two most urgent research priorities.^1^ Currently, these and other aspects of hypnozoite biology can be investigated experimentally only in part using the traditional *P. cynomolgi-*infected rhesus macaque model because of the inherent biological, economical, and ethical limitations. These limitations also restrict the number of molecules that can be tested for activity against the hypnozoite. Recently, hypnozoites were obtained in a long-term in vitro culture of *P. cynomolgi*-infected primary simian hepatocytes, opening the way to initiate investigations on the biological nature of the hypnozoites and to conduct cost-effective low- to medium-throughput screening of compounds to identify novel leads for radical cure.^10^,^11^ Similar in vitro, as well as in vivo, systems for drug screening and studying *P. vivax* hypnozoite biology are being developed.^12^,^13^

## Asexual Blood Stages and Merozoite Invasion

A distinct biological feature for *P. vivax* regarding its invasion and growth in host erythrocytes is its dependence on the Duffy blood group antigen expressed at the erythrocyte surface, although apparently Duffy-negative individuals can also become infected under some circumstances.

Until recently, it was commonly accepted that *P. vivax* merozoites could invade only erythrocytes expressing the Duffy blood group antigen (also known as Duffy antigen chemokine receptor [DARC]). This dependence on DARC explained why the geographic distribution of *P. vivax* coincided predominantly with tropical and temperate areas where the Duffy blood group is expressed on the surface of erythrocytes, which is the case for most human populations, except for sub-Saharan Africa. However, some recent data have challenged this belief. Evidence recently acquired in Madagascar, Kenya, Brazil, and elsewhere indicates that *P. vivax* can infect Duffy-negative individuals, albeit these infections may not be nearly as robust as in Duffy-positive individuals.^14^ Concern has been expressed that in areas with large interspersed populations of both Duffy-negative and Duffy-positive people, *P. vivax* might be evolving to infect human populations lacking the erythrocytic expression of DARC.^15^ Although this is presently only speculation, monitoring for *P. vivax* infections in Duffy-negative populations in Africa should be considered.^16^

The parasite ligand for DARC, Duffy binding protein (DBP), localized in the apical (invasive end) organelles of the merozoite, is a member of an intra- and interspecies family of invasion ligands known as Duffy binding protein–like (DBL) or, alternatively, erythrocyte binding protein–like (EBL) proteins. Most species of *Plasmodium* have multiple DBL/EBL family members in their genomes that mediate alternative pathways for invading red blood cells (RBCs). Until recently, sequencing of the genome of the Salvador I strain of *P. vivax* had indicated that DBP was the only DBL and thus likely no alternative invasion pathways existed to escape the lack of the DARC receptor. However, recent sequencing of genomes from other strains of *P. vivax* indicate there is a second duplicated DBP gene in the genomes of many isolates of *P. vivax*, observed originally in Madagascar^14^,^17^ and also throughout southeast Asia^17^ as well as a second DBL gene present and expressed in most strains, *P. vivax* erythrocyte-binding protein which, theoretically, might mediate invasion in Duffy-negative erythrocytes.^18^,^19^

DBP may be an important vaccine target as a cysteine-rich region mediates the specific binding of this invasion ligand to DARC. This binding domain, while mostly conserved, nevertheless exhibits considerable amino acid polymorphism outside the binding pocket while retaining its binding specificity. As a consequence, most blocking antibodies against the binding domain, while able to inhibit the interaction of DBP with DARC with homologous parasites and molecules, do not bind or show reduced binding to heterologous variants of DBP, although some immune individuals elicit antibodies that are able to cross this immunological barrier and inhibit DBP/DARC interactions for many different allelic variants of *P. vivax* DBP. Similar to apical membrane antigen-1-based vaccines for *P. falciparum*, vaccines based on DBP may need to contain several different allelic forms of the invasion ligand to be effective.^20^,^21^

The basis for preferentially invading reticulocytes is the presence in *P. vivax* merozoites of two large high molecular weight (> 300 kDa) proteins that bind specifically to reticulocytes, independently of DARC. These two invasion ligand proteins were originally termed reticulocyte binding protein 1 and 2 (RBP-1 and RBP-2). As with DBP and the DBL gene family, the RBPs are part of a large intra- and interspecies family of invasion ligands termed the RBP-like (RBL) proteins. Genome sequencing has revealed that there are at least six other RBL genes in the genome of *P. vivax* in addition to RBP-1 and RBP-2. However, most appear to be pseudogenes and likely not expressed, although transcripts might be detectable. RBP-1 is not very polymorphic with only three of 3,000 potential sites of non-synonymous changes noted. RBP-2c has a much greater number of non-synonymous mutations that produce the greatest number of amino acid substitutions, which could indicate that the RBP-2c ligand is important for reticulocyte invasion. It could also suggest allele-specific immune responses might be a challenge facing the development of RBL-targeting vaccines in the future. Recently, RBP-2a was shown to bind mature RBCs, and its crystal structure indicated structural similarity to the essential *P. falciparum* invasion ligand Rh5.^22^ Although the function(s) of the *P. vivax* RBL ligands are not fully understood, it is possible that these genes also serve as alternative pathways to invasion or for redundancy in the same pathway for invasion of reticulocytes.^21^,^23^,^24^

## Asexual Blood Stages and Diagnosis

Compared with *P. falciparum*, parasitaemia and pyrogenic threshold (the number of parasites that trigger a symptomatic malaria attack) are lower in *P. vivax* infections. The lower parasitaemia thus means that diagnosis and treatment are often delayed or missed entirely. This scenario is even more likely for relapse infections as parasite levels are lower and often less symptomatic with each successive relapse as a consequence of the gradual acquisition of anti-blood stage immunity. Consequently, prevalence is underestimated while transmission potential is enhanced, particularly as commitment to gametocyte production occurs early in the infection. Developing more sensitive diagnostic tools is a public health priority.

The strict preference for reticulocytes (generally around 0.5–1.5% of the total circulating erythrocytes) along with the relatively rapid acquisition of immunity explain the lower parasite levels observed during *P. vivax* infection, generally restricting peak parasite levels below 20,000 parasites per microliter of blood. This occurs even when reticulocyte levels increase in response to the profound anemia caused by high levels of destruction of mature uninfected erythrocytes during *P. vivax* infections.^25^

Vivax malaria has a lower pyrogenic threshold than *P. falciparum* infections in all groups of individuals such that fever and symptoms can occur with parasitemias undetectable by routine diagnostics: in a nonimmune host experiencing a *P. vivax* infection, the first febrile symptoms often occur 2−3 days before the parasites can be reliably detected in the peripheral blood by microscopy, though the parasites can be detected by polymerase chain reaction (PCR)–based assays. Data from Thailand indicate that the pyrogenic threshold for *P. vivax* (181 parasites/μL) is 8-fold lower than that for *P. falciparum* (1,460 parasites/μL), and data from PNG indicates a steeper relation between the fractions of fever attributable to *P. vivax* infections and parasite density than for *P. falciparum*.^26^,^27^ The lower pyrogenic threshold is reflected by higher concentrations of circulating tumor necrosis factor than for *P. falciparum* at equivalent peripheral parasitaemias.^28^ Cytokine production, endothelial activation, and pulmonary inflammatory responses are also higher during and after *P. vivax* infections compared with *P. falciparum* infections of similar parasite biomass. The discrepancy between higher cytokine levels and concomitant inflammatory responses on the one hand and milder disease on the other hand compared with *P. falciparum* remains unexplained.

The low parasitemia and pyrogenic threshold of *P. vivax* infections, particularly in patients with no or limited prior exposure, challenge accurate diagnosis by light microscopy and even more so by the currently available, sometimes less-sensitive rapid diagnostic tests (RDTs), which have a level of detection for *P. vivax* of 200−500 parasites/μL.

The problem of missing low parasite levels in endemic areas is less about missing clinical cases and more about transmission control, as it allows a larger pool of generally asymptomatic carriers who are often gametocytemic and potentially infectious to go undetected. The central diagnostic challenge is identifying the reservoir of *P. vivax* infections that must be reduced if *P. vivax* malaria is to be controlled and eliminated. It is becoming increasingly recognized that asymptomatic infections with low parasite densities are very common in areas of both high and low endemicity. In cross-sectional surveys in highly endemic PNG, 53–59% of all *P. vivax* infections were submicroscopic, and 93–96% of these were asymptomatic; in areas of lower endemicity, such as Flores, Indonesia, with an overall prevalence of 10.5%, nearly all (93%) were submicroscopic and none were with fever. Similar surveys in Brazil, Cambodia, Peru, and Vanuatu found submicroscopic infections to account for 67–89% of all infections (overall PCR prevalence: 2.4–14.7%), with the majority being asymptomatic. Furthermore, because of the presence of hypnozoites, the absence of blood-stage infections does not necessarily indicate that the person is free of the infection. In southeast Asia, *P. vivax* infections occur in 20–50% of patients soon after being treated for *P. falciparum*.^29^ Similarly, in three community-based pediatric cohorts in PNG, 61–100% of children who received presumptive treatment of only blood stages had recurrent *P. vivax* infection within 3 months. Irrespective of whether they were confirmed malaria positive by PCR at treatment, at least 60–80% of these infections were relapses.^30^,^31^

Both light microscopy and RDTs fail to identify the majority of *P. vivax* blood-stage infections, and there are no diagnostic tests that can identify hypnozoite carriers. Mass screening campaigns, even if based on a sensitive molecular diagnosis of blood-stage infection, may thus miss a large part if not the majority of people who have a recurrent *P. vivax* parasitemia within 3 months. Therefore, mass drug administration (MDA) with both an effective anti-blood-stage as well as an anti-liver-stage drug may be one of the few ways of attacking the *P. vivax* “reservoir” decisively.^31^,^32^

## Sexual Blood Stages and Transmission

Infectious gametocytes appear in the circulation almost simultaneously with asexual blood stages within a few days before or after patency in *P. vivax* infection, and often concurrently with relapse episodes. This extends the transmission potential before or in the absence of treatment.

**Figure 3.**
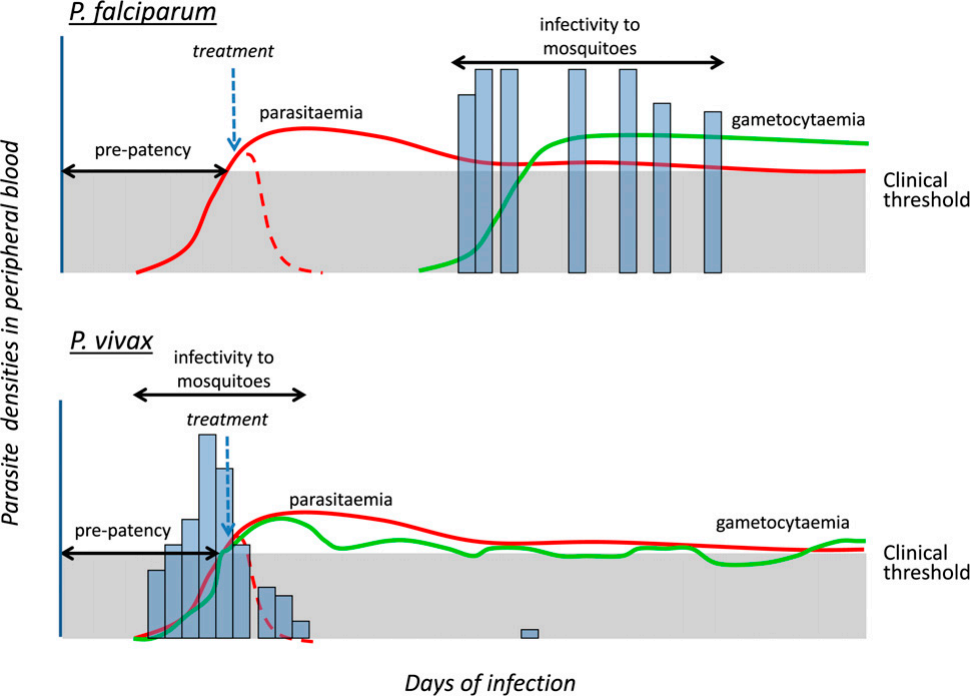
Schematic representation of the course of a blood infection with time in *Plasmodium falciparum* (**A**) and *Plasmodium vivax* (**B**). The vertical axis is parasite densities (sexual and asexual forms) in the blood; the horizontal axis is time since infection; both are arbitrary and there are no units. In the absence of treatment, asexual blood parasitaemia is depicted in solid red line and gametocytemia in solid green lines. The bars represent infectivity of gametocytes to mosquitoes. The clinical threshold parasitaemia is shown below the gray area; symptoms tend to occur at lower parasitemias in a *P. vivax* compared with *P. falciparum* infection. In *P. vivax* gametocyte counts are generally one-tenth of the asexual parasite counts. When effective treatment is administered at the point indicated by the vertical arrow, the parasitemia declines as depicted by the broken red lines preventing further gametocytemia. Thus early and effective treatment will, in *P. falciparum*, abolish gametocytes and infectivity to mosquitoes, whereas in *P. vivax* transmission has already occurred by the time treatment is administered.

There are fundamental differences between *P. vivax* and *P. falciparum* infections with respect to the appearance of transmission stages. While in *P. falciparum* the infectious gametocytes only appear in the peripheral blood circulation late in an acute infection, generally 10−14 days after the onset of clinical symptoms, they appear earlier in primary *P. vivax* infections within a few days of patency and concurrently with patency in relapse episodes. Therefore, *P. falciparum* cases are unlikely to have transmitted the infection before the initiation of treatment, but this is not necessarily so for many *P. vivax* cases. Thus, early diagnosis and treatment of *P. vivax* may not prevent transmission as it usually does quite effectively for *P. falciparum*. Therefore it is very important that vector control measures are implemented if *P. vivax* is to be effectively controlled^33^,^34^ (see Figure 3

).

*Plasmodium vivax* gametocytes are observed in the majority of clinical infections, at densities of up to 10% those of asexual parasites, and circulate for a maximum of 3 days. There is a weak association between *P. vivax* gametocyte density and mosquito infection rates. Malaria therapy patients who were infected with *P. vivax* were infectious to mosquitoes as soon as asexual parasites were present in the blood film. This was well before the appearance of microscopically detectable gametocytes, and such patients remained infectious for several weeks after clinical symptoms had resolved and gametocytemia dropped below 10/μL.^35^ This infectivity of low or undetectable gametocyte densities has been confirmed by numerous studies of naturally infected patients.

A number of studies have used molecular methods to detect gametocytes, based on Pvs25 mRNA. These studies found a greatly increased gametocyte detection rate compared with microscopy and demonstrated that 50% to > 90% of all PCR-detectable *P. vivax* infections had detectable levels of gametocytemia.^36^,^37^ Another study found a better correlation between gametocyte density and intensity of infection in mosquitoes with a molecular tool for *P. vivax* gametocyte detection compared with microscopy.^38^,^39^

This biological difference in the transmission modalities of the two parasites may help explain the limited spread of drug resistance in *P. vivax*. Resistance to chloroquine was first reported for *P. falciparum* in the 1950s, and it spread rapidly over the following decades to all endemic regions. A similar, often faster pattern, of emergence and spread of *P. falciparum* resistance to other antimalarial drugs has also occurred. By contrast, the first report of chloroquine resistance in *P. vivax* was in 1989 from PNG,^40^,^41^ and this resistance appears to have spread at much slower pace, with only isolated reports of chloroquine resistance from other parts of the world. In a drug-sensitive *P. falciparum* blood infection, early treatment will significantly reduce infectivity to mosquitoes. Should drug-resistant mutants have been selected, the gametocytes that may subsequently arise will carry and spread these mutations. In *P. vivax* on the other hand, transmission starts before exposure to the drug. Thus, theoretically, in persons where wild-type and mutant parasites occur, the drug-sensitive parasite will not be at a disadvantage with respect to transmission, and consequently the selective pressure for drug resistance will be reduced.^33^

## Mosquito Stages and Vectorial Capacity

Both in the past and currently, the geographic range of *P. vivax* prevalence substantially exceeds that of *P. falciparum*, extending into the high latitude temperate zones. The primary reason for this is the ability of the parasite to complete its development cycle at lower temperatures and more quickly than *P. falciparum*.

The geographical range of malaria parasites is determined by the availability of susceptible anophelines and of environmental conditions to allow for the time it takes for sporozoites to form and reach the salivary glands following an infectious blood meal. Although *P. vivax* is currently found mainly in tropical regions,^42^ as is *P. falciparum*, its endemic and epidemic transmission was previously common throughout the temperate zones during summer months (including the short subarctic summer), and these areas remain susceptible to its reintroduction.^43^–^45^

While many different species of anopheline mosquitoes can be found in nearly all regions of the world, only a subset are susceptible to successful infection by *Plasmodium* parasites. Furthermore, the mean duration for the full development of *P. vivax* in the mosquito is shorter (8–16 days) than it is for *P. falciparum* (9–22 days), and can be achieved at an average temperature of 16°C, whereas the lowest limit for *P. falciparum* is 18°C.^46^

This explains why vector control measures deployed primarily for *P. falciparum* have much less impact on the transmission of *P. vivax*, as they must cover a broader range of species (behaviors, breeding sites) to be effective for the latter. Given the extended duration of the *P. vivax* infection (as a result of relapses) and the high transmission potential (early gametocyte production), it becomes imperative that entomological surveys are specifically conducted to tailor vector control measures to *P. vivax*.

## Immunity to Vivax Malaria

In highly malaria-endemic areas, the burden of disease of vivax malaria occurs earlier in life and drops earlier (after the first 2–3 years) than falciparum. This is as a result of more rapidly acquired clinical immunity (acquired by ∼5 years of age) controlling blood-stage parasite densities (often to a level only detected by PCR), rather than the acquisition of a significant immunity against infection per se. Conversely, the prevalence of infections detected by PCR increases with age for both parasites. However, the molecular force of infection (i.e., the incidence of genetically distinct infections) is higher for vivax malaria and is largely caused by relapse.

It has long been known that *P. vivax* is a disease that predominantly affects children at a younger age than *P. falciparum*. Studies in areas, which are highly endemic for both *P. falciparum* and *P. vivax* such as the island of New Guinea, but also the western border of Thailand, Sri Lanka, and Vanuatu show that 1) *P. vivax* is the predominant source of malaria infections and disease in children < 2 years of age; 2) the incidence of *P. vivax* malaria then decreases rapidly from the second year of life, whereas *P. falciparum* incidence continues to increase until the fourth year; and 3) children 5−14 years of age have acquired an almost complete clinical immunity to *P. vivax* but remain at considerable risk of *P. falciparum* illness despite a similar burden of blood-stage infections (reviewed in Ref 47). As a consequence of this remarkable difference in the rate of acquisition of clinical immunity, in New Guinea the incidence of *P. vivax*-attributable febrile illness in routine health surveillance is highest in children 1–2 years of age, while that of *P. falciparum* peaks in children 2–4 years of age. Although *P. vivax* is a very significant source of severe illness in infants, the proportion of *P. vivax* infections presenting with severe symptoms decreases rapidly with age, and in children aged > 1 year, the incidence of severe *P. vivax* illness is significantly lower than that caused by *P. falciparum*.

Similar patterns are found in the age-specific prevalence of infection when diagnosed by light microscopy; conversely, when infections are diagnosed by PCR, the prevalence of both *P. vivax* and *P. falciparum* infections increase overall, and the peak prevalence shifts into older age groups.^48^ This means that submicroscopic, sub-patent parasitemia is persistent even in older age groups. It has been estimated that relapses from long-lasting liver stages account for between 60% and 80% of the burden of *P. vivax* blood-stage infections,^30^,^31^ with relapses caused by genetically distinct but often related parasite clones.^6^,^7^ This explains why the incidence of newly acquired, genetically distinct, *P. vivax* blood-stage infections (i.e., the molecular force of blood-stage infection) is generally higher than that of *P. falciparum*, independent of the entomological inoculation rate.^49^

The age at first exposure may be an important modulator of the natural acquisition of malarial immunity. Studies involving nonimmune migrants to Indonesian New Guinea who were exposed for the first time to heavy transmission indicated that adults acquired clinical immunity to malaria after relatively few *P. falciparum* infections, in contrast to children, who remained susceptible after comparable exposure. This relationship was not observed with *P. vivax* infections; adults did not acquire clinical immunity any more quickly than children.^50^

## Pathogenesis in Vivax Malaria

Although previously believed to be a benign infection, *P. vivax* is now well recognized as a significant cause of disease in all endemic areas. In addition, mortality occurs in *P. vivax*, possibly related to cytoadhesion or other pathogenic mechanisms different from those operating in *P. falciparum*.

All stages of *P. vivax* can be routinely found in blood smears, whereas erythrocytes infected with trophozoites and schizonts of *P. falciparum* adhere to vascular endothelium and thus are rarely found in the peripheral blood, except in overwhelming infections.^51^ As noted above, *P. vivax* prefers to invade reticulocytes, and a reason for such preference may be a physiological and metabolic necessity for growth and survival in the host's circulating erythrocytes. *Plasmodium vivax* and related sibling species are not known to produce cytoadhesive variant ligands at the surface of infected erythrocytes as *P. falciparum*-infected erythrocytes do. These ligands bind endothelial receptors to immobilize the rigid infected RBCs to prevent their destruction in the spleen or while passing through capillaries. In contrast, *P. vivax-*infected mature erythrocytes are highly flexible and will pass through capillaries and splenic fenestration quite easily. The reticulocyte is not yet discoid and as rigid like a mature erythrocyte, which may aid in keeping the reticulocyte flexible.^52^,^53^

Thus, the two species have obviously evolved different strategies to escape removal by the spleen: sequestration in the microcirculation during advanced stages of maturation to reduce removal by the spleen for *P. falciparum* versus increased flexibility of young erythrocytes to pass unhindered through splenic sinusoids for *P. vivax*. The flexibility of infected reticulocytes may be further increased by the parasite-induced caveola-vesicle complexes in the infected reticulocyte membrane (appearing on microscopic examination of blood films as punctate spots known as Schüffner's dots or stippling) and other parasite-induced modifications of the host-cell cytoskeleton.^54^ The preference for reticulocytes has also prevented or hindered so far the development of continuous in vitro culture of *P. vivax* parasites.

The absence of cytoadherence and sequestration by *P. vivax* also explains some of the differences in pathogenesis and clinical features between *P. falciparum* and *P. vivax*, and the relative lack of severe manifestations like coma and organ failure in the latter species. However, *P. vivax* is not as avirulent a parasite as its nickname “benign tertian malaria” would suggest, and may cause severe disease in some patients.^55^ The symptoms of a normal uncomplicated course of infection are very harsh and debilitating.^27^,^56^ Furthermore, there is growing evidence of severe and fatal disease syndromes despite low levels of parasitemia. Although *P. vivax* is not generally thought to sequester in the vasculature, it is possible that severe *P. vivax* disease results when the bulk of biomass is hidden in the bone marrow or spleen.^57^,^58^

## Genetic Diversity of *P. vivax*

*Plasmodium vivax* is more genetically diverse than *P. falciparum*, potentially making it a more complex target for therapeutic intervention. The higher genetic diversity is also indicative of this species' relatively stable transmission and resilience against population bottlenecks after intervention.

Several studies have demonstrated that *P. vivax* is a more genetically diverse parasite than *P. falciparum* both with collections of isolates from different locations worldwide and with field samples from co-endemic regions^59^–^66^ (Table 2 ). In most countries studied outside of sub-Saharan Africa, *P. vivax* is the predominant infection in the community and at similar or higher prevalence among clinical cases. This would partly explain the higher diversity, since under these circumstances a much higher effective population size would be expected. However, in PNG, where *P. falciparum* prevalence was slightly higher, *P. vivax* also had substantially higher diversity.^64^ In addition, genomic sequencing of *P. vivax* populations has demonstrated that within population diversity of *P. vivax* is higher than that of a small panel of *P. falciparum* isolates from disparate geographic regions.^66^ Moreover, while the extent of *P. falciparum* diversity is tightly correlated with regional levels of transmission,^67^ *P. vivax* harbors high genetic diversity even in low-transmission areas.^68^ High diversity provides greater evolutionary potential to evade host immunity, develop drug and vaccine resistance, and adapt to different human and anopheline hosts. There is an apparent contradiction between this high genetic diversity and the fact that *P. vivax* immunity develops more quickly than to *P. falciparum*, an overall less diverse parasite. This may be related to a number of factors: 1) *P. vivax* lacks a homologue of *Plasmodium falciparum* erythrocyte membrane protein and thus is not thought to exhibit switching in clonal expression of variant surface antigens; 2) a single infective mosquito bite results in one *P. falciparum* but five *P. vivax* blood-stage infections (one primary and four relapses^31^), as a result PNG children experience approximately 3 times more distinct *P. vivax* blood stage infections^37^, despite comparable entomological inoculation rates for both species; and 3) a primary *P. vivax* infection and its relapses are generally diverse but genetically related,^7^ thus results both in exposure to known antigenic variants and boosting of allele-specific responses. The link between genetic and antigenic diversity of malaria parasites remains a major gap in knowledge despite its key relevance to malaria vaccine development.

A better understanding of *P. vivax* diversity at different levels of transmission would enable control programs to mount appropriate control strategies and help monitor whether an intervention is making an impact. Genetic diversity, generated through cross-mating between distinct strains after co-transmission to the mosquito from hosts infected with more than one strain, is a sensitive marker for *P. falciparum* transmission.^62^ However, since *P. vivax* has high levels of diversity irrespective of the intensity of transmission, this relationship is not well defined. Comparative studies of the population genetic structure of the two species demonstrate a less structured population for *P. vivax*, indicating higher levels of gene flow between geographic areas.^60^,^61^ A major reason for the higher diversity and limited genetic structure of *P. vivax* populations may be relapse, providing greater potential for multiple infection^65^ and thus, cross-mating and further dissemination than *P. falciparum*.^66^ At low transmission intensities, increasing linkage disequilibrium has been observed indicating more frequent transmission of highly inbred strains. In the event of sustained low transmission, relapses would be more likely to derive from the same inoculum, thus eventually reducing diversity and increasing population structure.

It is unclear whether the ability to maintain high diversity at low transmission for an extended period affords the parasite population greater resilience against interventions. Reports of *P. vivax* treatment failure rapidly emerged after the introduction of sulfadoxine–pyrimethamine in PNG, yet *P. vivax* chloroquine resistance emerged much more slowly and lagged far behind that of *P. falciparum*. Less exposure to drugs and higher rates of multiple infection allowing recombination to breakdown any multilocus resistance haplotypes may have slowed the emergence of resistance. High diversity may also make the development of vaccines against *P. vivax* challenging. However, the exact relationship between genetic diversity and antigenic diversity and immunity in malaria is likely to be quite complex. High genetic diversity of *P. vivax* may reflect a more ancient parasite population rather than adaptation to host immune responses. This is supported by the earlier immunity described above suggesting that immune targets in *P. vivax* are more antigenically conserved.^32^ Studies investigating the dynamics of polymorphism in *P. vivax* antigens will be essential to dissect the contribution to immune escape. In addition, antimalarial interventions incorporating drugs or vaccines will need to monitor parasite populations closely for the emergence of resistant strains.

## Conclusions

This article summarizes our current understanding of the biology of *P. vivax* and the specific aspects that make it a different parasite from *P. falciparum*. The main characteristics of vivax malaria and their consequences and implications for research and control are summarized in Table 1. These differences explain why *P. vivax* is relatively unaffected by interventions tailored to control *P. falciparum*. The article further suggests knowledge gaps that deserve more investment to resolve key questions that could help identify improved and better-tailored interventions, including transmission control, diagnosis, and treatment.

The ability to develop at lower temperature and over a shorter sporogonic cycle in the vector means that *P. vivax* extends beyond tropical climates into temperate regions and that it is less susceptible to vector control measures such as indoor residual spraying, which reduce mosquito' life span and have proven effective against *P. falciparum*.

Having dormant forms in the liver (hypnozoites) means that one successful infection will generate a number of parasitological and clinical episodes without reinfection. Therefore, recurrent cases cannot be prevented via vector control, though, paradoxically, successful transmission control could reduce the burden of disease of vivax malaria more than for *P. falciparum*, because avoiding one infection will result in preventing a number of clinical episodes over several years. Eradicating the infection remains at best a long-term goal, until a drug effective against the hypnozoite that is also safe for wide deployment becomes available; today we only have 8-aminoquinolines, whose use is limited by toxicity in glucose-6-phosphate dehydrogenase deficient populations. The current lack of reproducible in vitro and in vivo models makes it very difficult to identify new compounds; further investments are needed to develop adequate models.

Vivax malaria is diagnosed late, because infected people get ill with low parasite densities, which cannot be detected with current diagnostics, such as RDTs and microscopy, that work for *P. falciparum* because of *P. vivax*'s preference for reticulocytes and its low pyrogenic threshold. Delayed diagnosis means not only delayed treatment (hence prolonged morbidity, especially anemia) but also ability to transmit over an extended period. This is further amplified by the fact that mature gametocytes appear simultaneously with asexual forms—hence transmission occur before diagnosis and treatment. Investment in new, more sensitive diagnostics adapted to *P. vivax* is needed.

To determine the importance of low-level, asymptomatic *P. vivax* infections in sustaining transmission, it is essential that further studies into the infectious reservoir; the association of parasitemia, gametocytemia, and infectivity; and thus the *P. vivax* effective population size are conducted in areas with variable transmission intensities in different vivax-endemic areas.

*Plasmodium vivax* has higher genetic diversity than *P. falciparum*, even in areas of low transmission, which makes it a more complex infection to study with respect to both transmission, immunity and response to treatment. This diversity also renders genetic markers less useful for *P. vivax* epidemiology than they are for *P. falciparum*, especially because we have a very limited understanding of the relationship between *P. vivax* diversity and transmission.

## Figures and Tables

**Table 1:** Main characteristics of *Plasmodium vivax* biology and their implications

Characteristic	Consequence	Implications for control	Implications for research
Hypnozoites: dormant parasites in the liver	Relapses: (a) generate parasitological and clinical episodes without reinfection, (b) help maintain genetic diversity	Radical cure requires elimination of hypnozoites. Only 8-aminoquinolines available to date but use limited by toxicity	Need to develop reproducible in vitro and in vivo models to understand mechanisms of quiescence and activation
		No diagnostic to identify subjects with potential for relapses	Need to discover and test new medications for radical cure
		Recurrent cases cannot be prevented via vector control	Need to develop diagnostics for liver-stage parasites
Asexual blood stages: preference for reticulocytes and low pyrogenic threshold	Morbidity with low parasite densities, and parasitaemia undetectable with conventional methods	Difficult to diagnose with rapid diagnostic tests or microscopy	Need to develop continuous in vitro culture systems
		Delayed diagnosis and treatment (especially relapsing episodes)	Need to develop new, more sensitive diagnostics for blood-stage parasites
	Anemia	Protracted transmission	
		Prevalence underestimated	
Mature gametocytes appear almost simultaneously with asexual forms	Transmission occurs before diagnosis, and treatment on occasion of primary infection and relapses	Early treatment cannot prevent transmission for current episode	
	Resistance is not spread as efficiently as in *Plasmodium falciparum*	Entomological surveys required to tailor vector control measures	
Vectorial capacity: can complete its development cycle in the mosquito at lower temperature and more quickly than *P. falciparum*	(a) Larger endemic geographic range into temperate region	(a) Not confined to tropical climates	
	(b) Less susceptible to vector control measures that reduce mosquitos' life span	(b) Vector control via indoor residual spraying may be not as effective as for *P. falciparum*	
Immunity	Clinical immunity acquired earlier in life	Burden of disease in young children in high-transmission areas	More studies needed to improve understanding of immunity to *P. vivax* vs. *P. falciparum*
	Higher molecular force of infection caused by relapses independent of inoculation rate	Rate of infections increases with age	
Pathogenesis	High flexibility of reticulocytes allows escaping splenic removal without sequestration: relative lack of severe manifestations	Burden of disease underestimated	Develop better surveillance tools to more accurately map vivax burden of disease
	However, significant morbidity (debilitation, anemia) and evidence of severity and fatality		
Higher genetic diversity	Association with epidemiology not yet clear	More stable transmission	More studies needed in a range of transmission settings
	Sustained at low transmission	More difficult to control	More complex vaccine target

**Table 2:** Studies comparing *Plasmodium falciparum* and *Plasmodium vivax* genetic diversity

Region	*Plasmodium* isolates included	Dominant infection	Data type	Higher *P. vivax* diversity	Reference
Venezuela	30 *P. falciparum* and 73 *P. vivax* isolates	*P. vivax*	*Ama1* SNPs	Yes	59
Worldwide reference isolates	5 isolates per species	Variable	Whole genome SNPs	Yes	60
Venezuela	108 *P. falciparum* and 107 *P. vivax* field isolates	*P. vivax*	Microsatellites	Yes	61
Vanuatu	165 *P. falciparum* and 100 *P. vivax* field isolates	*P. vivax*	*msp1* and *csp1* SNPs	Yes	62
Cambodia	164 *P. falciparum* and 87 *P. vivax* field isolates	*P. vivax*	Microsatellites	Yes	63
Papua New Guinea	308 *P. falciparum* and 193 *P. vivax* field isolates	*P. falciparum*	Microsatellites	Yes	64
Indonesia	166 *P. falciparum* and 168 *P. vivax* clinical isolates	*P. vivax*	Microsatellites	Yes	65
Worldwide clinical isolates	12 *P. falciparum* and 220 *P. vivax* clinical isolates	Variable	Whole genome SNPs	Yes	66

SNPs = single nucleotide polymorphisms.
